# FAPI PET/CT in Diagnostic and Treatment Management of Colorectal Cancer: Review of Current Research Status

**DOI:** 10.3390/jcm12020577

**Published:** 2023-01-11

**Authors:** Zhiming Cheng, Shu Wang, Shuoyan Xu, Bulin Du, Xuena Li, Yaming Li

**Affiliations:** Department of Nuclear Medicine, The First Hospital of China Medical University, Shenyang 110001, China

**Keywords:** colorectal cancer, FAPI, FDG, PET, SUV_max_, SUV_mean_

## Abstract

FAPI PET/CT is a novel imaging tool targeting fibroblast activation protein (FAP), with high tumor uptake rate and low background noise. Therefore, the appearance of FAPI PET/CT provides a good tumor-to-background ratio between tumor and non-tumor tissues, which is beneficial to staging, tumor description and detection. Colorectal cancer has the biological characteristics of high expression of FAP, which provides the foundation for targeted FAP imaging. FAPI PET/CT may have a potential role in changing the staging and re-staging of colorectal cancer, monitoring recurrence and treatment management, and improving the prognosis of patients. This review will summarize the application status of FAPI PET/CT in colorectal cancer and provide directions for further application research.

## 1. Introduction

Colorectal cancer (CRC) is a malignant tumor with a high incidence and mortality around the world [1], and about 20% of patients have metastasis at the first diagnosis, which seriously endangers human health. Early diagnosis and definite staging can help to prolong the life expectancy of more colorectal cancer patients and reduce unnecessary treatment [2,3]. The application of positron emission tomography can obtain multiple biomarkers in vivo, providing visual evidence for disease management and treatment strategy for colorectal cancer [4,5,6].

As a membrane anchored peptidase highly expressed in cancer-associated fibroblasts (CAFs) in more than 90% of epithelial tumors, fibroblast activating protein (FAP) can lead to the progression of different cancers. The uptake of various tracers targeting FAP-labeled fibroblast-activating protein inhibitors (FAPI) is high in most tumors but low in normal tissues, such as in the brain and abdomen, resulting in a good tumor-to-background ratio (TBR) and good tumor delineation. Compared to FDG PET/CT, FAPI PET/CT has more advantages in detecting primary and metastatic lesions. In addition, targeting FAP imaging may break new ground with its applications, such as non-invasive tumor characterization, tumor staging and tumor treatment effect monitoring [7]. This article reviews the current research status of FAPI PET/CT in diagnosis and management of colorectal cancer (Table 1).

## 2. Fibroblast Activation Protein (FAP) and FAPI

FAP is an atypical type II transmembrane serine protease with dipeptidyl peptidase (DPP) and endopeptidase activities [8]. FAP is highly expressed on activated fibroblasts, and fibroblasts undergo dimerization and glycosylation when activated [9]. In adult tissues, activated FAP almost only exists in the stroma of wound healing, fibrosis, and malignant tumors [10]. FAP participates in epithelial–mesenchymal transition (EMT) in malignant tumors, promotes the invasion and migration of tumor cells, and ultimately promotes tumor metastasis [11], which indicates that CAFs play an important role in the development of tumors. Compared with normal fibroblasts, the expression of FAP in CAFs has relative specificity. In histopathological studies, FAP-positive CAFs have been found in more than 90% of epithelial tumors, so FAP has become a therapeutic and imaging target for various malignant tumors [12,13,14]. The accuracy and effectiveness of PET probe diagnosis and treatment depend on targeting specificity and tumor-specific uptake [15].

By using chelators to connect FAP inhibitors (FAPI) or FAP antibodies to radionuclides and then performing PET/CT imaging, PET/MR imaging or SPECT imaging, the imaging research of malignant tumors targeting FAP was carried out [16,17,18]. The imaging agent may be used to determine the clinical stages and monitor the recurrence of cancer and may be used as a biomarker or patient selection tool in clinical trials to predict clinical treatment response.

## 3. Colorectal Cancer and CAFs

Colorectal cancer accounts for about 10% of cancer and cancer-related deaths diagnosed worldwide each year [1,19]. Colorectal cancer is a disease influenced by many factors, such as genetics, the environment and diet. Most colorectal cancer is caused by genetic or epigenetic changes in stem cells or stem cell-like cells, which inactivate tumor suppressor genes and activate oncogenes, and aberrant foci gradually develop into adenoma and cancer [20,21,22].

CRC is a complex tumor which consists of tumor stroma or tumor microenvironments composed of other cells and non-cellular components in addition to tumor cells. Cellular components include immune cells at different activation stages, vascular cells and mesenchymal cells mainly composed of fibroblasts [23,24,25]. In the early stages of CRC, CAFs are activated, accumulate, and exert a strong response to promote the proliferation of connective tissue. Over 90% of the total tumor masses are composed of CAFs and their fibrosis gradually develops into cancer through co-transformation with stem cells. The CAFs are key components of tumor stroma, which can support immune suppression microenvironment; promote the growth, invasion and angiogenesis of colorectal cancer cells; and are closely related to the poor prognosis of patients [26,27,28,29]. Studies have shown that the removal of tumor stroma can improve delivery of therapy or systemically applied radiation and enhance the immune response of the body [30]. Therefore, CAFs play an important role in the occurrence and development of CRC. Because there are many CAFs in the extracellular matrix of colorectal cancer and CAFs already exist in the early stage of colorectal cancer metastasis, it is possible to detect the expression of CAFs in colorectal cancer via special methods so as to determine the tumor size and metastasis of colorectal cancer and provide powerful evidence for determining the early clinical staging, re-staging, and monitoring recurrence of colorectal cancer. In colorectal cancer, FAP is characterized by over-expression in tumor cells and tumor stroma, and its expression level is a sign of the early development of CRC. Increased expression level of FAP is related to the poor prognosis, high grade and invasion of CRC [31,32,33]. Furthermore, FAP is a promising therapeutic target for colorectal cancer. It is reported that colorectal cancer can be treated by ^90^Y-FAPI46 [34]. Therefore, FAP represents a highly interesting target for CRC diagnosis and treatment applications, FAPI PET/CT targeting FAP can provide imaging basis for CRC staging, re-staging, and monitoring recurrence.

## 4. FAPI PET/CT Imaging

### 4.1. FAPI PET/CT Imaging in Normal Biological Organs

Good TBR is an important factor that affects image quality, and it is also an important prerequisite for disease diagnosis. Previous studies have reported the biodistribution of ^68^Ga-labeled FAPI imaging agents in various normal organs in human body imaging. The results showed that the FAPI tracer rapidly reached its stable physiological biodistribution. Its uptake could be detected as early as 10 min in normal organs, primary tumors and metastatic foci, and the visible image could be maintained for 3 h. The uptake of imaging agent in the liver decreased over time (SUV_max_ at 10 min vs. SUV_max_ at 3.3 h: 7.4 vs. 5.0). In contrast, tracer uptake in tumor tissues is very fast, and retention amount is higher than in normal organs (SUV_max_ at 10 min vs. SUV_max_ at 3.3 h: 15.5 vs. 13.4). Catherine Meyer et al. observed the highest TBR in bone marrow, with a ratio of 31 at 3.3 h. The ratio of tumor to muscle was 10.7 at 10 min and more than doubled to 22.8 at 3.3 h. At 3.3 h, the next highest TBRs were observed in the heart (19.1), spleen (18.9), and liver (16.8) [35], indicating that the maximum TBR in the lesion steadily increases as background activity decreases. Radioactive tracers in non-target organs are washed out imperceptibly and quickly removed, thus reducing the radiation load in normal organs. Stable clearance from radioactive blood pool and a rapid accumulation of activity were observed in the bladder, and the activity doubled at 60 min, which indicated that the kidney cleared quickly and produced high contrast images [36].

The uptake of FAPI is low in normal tissues. Giesel et al. reported that ^68^Ga-FAPI uptake was lower than ^18^F-FDG uptake in brain parenchyma (^68^Ga-FAPI, 0.09 vs. ^18^F-FDG, 10.72), oral mucosa (^68^Ga-FAPI, 2.04 vs. ^18^F-FDG, 3.33), parotid gland (^68^Ga-FAPI, 1.71 vs. ^18^F-FDG, 2.04), myocardial (^68^Ga-FAPI, 1.50 vs. ^18^F-FDG, 3.27), blood pool (^68^Ga-FAPI, 1.81 vs. ^18^F-FDG, 2.34), liver (^68^Ga-FAPI, 1.42 vs. ^18^F-FDG, 3.10) and pancreas (^68^Ga-FAPI, 1.82 vs. ^18^F-FDG, 1.99), spleen (^68^Ga-FAPI, 1.33 vs. ^18^F-FDG, 2.60), and kidney cortex (^68^Ga-FAPI, 2.20 vs. ^18^F-FDG, 2.80) [37]. Because the uptake of FAPI by normal brain and liver tissue is obviously lower than that of FDG, FAPI creates better conditions for imaging brain and liver tumors. Therefore, FAPI may be better at detecting metastatic lesions of malignant tumors (such as brain metastases and liver metastases), and the detection effect of FAPI may be superior to that of ^18^F-FDG. For the gastrointestinal tract, FAPI did not show obvious absorption (measured in colon transversum: mean SUVmax 1.40 vs. 2.05). When gastrointestinal tumors occur, good contrast is more conducive to the description of malignant tumors [37,38].

Because of its low intake and high contrast in normal tissues, FAPI PET/CT can show the pathological areas characterized by fibrosis well. FAPI PET/CT may have unique advantages in tumor or non-tumor lesions with fibrosis occurring in brain parenchyma, heart, spleen, liver and pancreas tissue, but further studies are necessary to prove it.

### 4.2. FAPI PET/CT Features of Primary and Metastatic Colorectal Cancer

**Figure 1:**
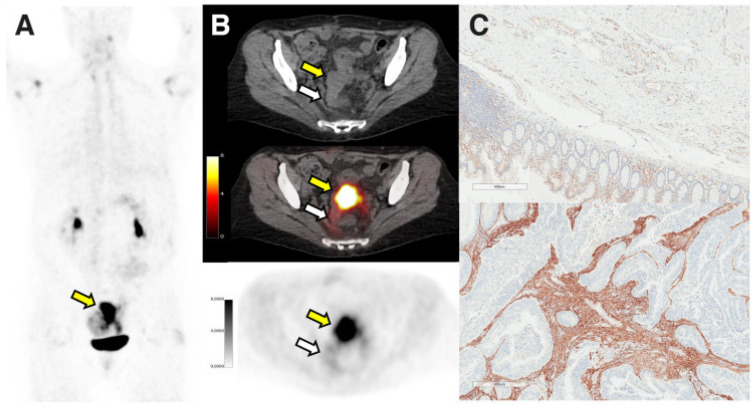
Figure shows the strong uptake of sigmoid adenocarcinoma in ^68^Ga-FAPi-46 PET/CT (SUV_max_, 15.9; SUV_mean_, 12.8). The yellow arrow shows the adenocarcinoma of the sigmoid, and the white arrow shows the surgically removed normal area (PET maximumintensity projection (**A**), axial CT ((**B**), top), axial PET/CT [B, middle], and axial PET [B, bottom]). And immunohistochemistry shows that FAP demonstrated absent to weak expression in normal tissue ((**C**), top) and was strongly expressed in intratumoral and peritumoral stroma ((**C**), bottom). Adapted from Ref. [39].

According to a relevant study [39], the SUV_max_, SUV_mean_ and FAP immunohistochemistry scores of ^68^GA-FAPI-46 were higher in various cancer tissues than in adjacent non-cancer tissues: the SUV_max_ (7.7 vs. 1.6); the SUV_mean_ (6.2 vs. 1.0); the FAP immunohistochemistry score (2.8 vs. 0.9). The cancer types with the highest uptake rates were pancreas, stomach, colon, and uterus. FAP immunohistochemistry score was positively correlated with ^68^Ga-FAPI-46 SUV_max_ (*r* = 0.781 [95% CI, 0.376–0.936], *p* < 0.001) and SUV_mean_ (*r* = 0.783 [95% CI, 0.379–0.936], *p* < 0.001) in cancer tissues [39]. In addition, studies have shown that the expression level of FAP in CRC is positively correlated with the consensus molecular subtype 4 (CMS4) of treatment resistance and poor prognosis factor, and FAPI PET/CT is valuable for comprehensive evaluation of CMS4 in CRC [40]. Therefore, ^68^Ga-FAPI-46 shows high metabolic uptake in tumor tissues, which can reflect the expression quantity of FAP in tumor lesions and provides a new means and method for non-invasive imaging and targeted FAP therapy of solid tumors. In a study on ^68^Ga-FAPI uptake of 28 different cancers, it was found that colorectal cancer had intermediate FAPI uptake, and SUV_max_ was between 6 and 12 [41]. A 56-year-old sigmoid adenocarcinoma patient (ybT4bN0Mo) underwent ^68^Ga-FAPI-46 PET/CT imaging before operation, and immunohistochemical staining was performed after operation. The results showed that the area corresponding to the resected tumor showed intense uptake of ^68^Ga-FAPI-46 (SUV_max_, 15.9; SUV_mean_, 12.8), which was consistent with the results of immunohistochemistry and shows that FAP is strongly expressed in the stroma inside and around the lesions [39] (Figure 1).

The liver is the primary target organ for colorectal cancer metastasis. Because of its low background degree of FAPI, the liver has a good TBR, which may be helpful to detect liver metastases [38]. The existence of liver metastases is very important for the treatment plan and survival rate of colorectal cancer [42,43]. As early as 1994, FAPI was recognized as a potential molecular probe target, and the TBR can reach 21:1, which is more favorable for the detection of occult liver metastases. At that time, antibody ^131^I-mAb-F19 was used to detect liver metastasis of colorectal cancer less than 1 cm [44]. This means that tumor mesenchymal cells with high expression of FAP exist in early liver metastases of colorectal cancer and can be detected by FAPI PET/CT, which may make up for the lack of obvious FDG uptake caused by the small number of tumor cells in early liver metastasis. In the study of monitoring liver metastasis of colorectal cancer, it was found that ^68^Ga-FAPI PET/CT could detect more true positive patients (12 cases vs. 10 cases) than ^18^F-FDG PET/CT, and their sensitivities to liver metastasis were 96.6% and 70.8%, respectively [45]. Clemens Kratochwil et al. used PET/CT to detect high FAPI uptake of less than 1 cm in the liver and lung, and the diagnosis of metastatic colon cancer was confirmed [41]. A study of 28 cases of malignant tumors in the lower digestive tract showed for the first time that FAPI showed high uptake and good TBR in primary and metastatic colorectal cancer lesions, which further changed the clinical stage of TNM in related patients and improved target volume delineation [46]. For the lymph node metastasis of colorectal cancer, the present research shows that there are many stroma in the lymph nodes and corresponding lymph node metastases (LNM) of CRC, which indicates that FAPI PET/CT may improve the identification of lymph node metastases of CRC [47].

^68^Ga-FAPI PET/CT showed intermediate ^68^Ga-FAPI uptake in primary colorectal cancer lesions (SUV_max_, 8.6). The SUV_max_ and SUV_mean_ of FAPI in metastatic lesions were 7.95 (±3.49) and 3.96 (±1.92), respectively. At the same time, the activity of background and normal organs was very low (muscle, SUV_max_:1.60 and SUV_mean_: 1.11; blood pool, SUV_max_:1.85 and SUV_mean_: 1.21; normal liver parenchyma: SUV_max_: 1.46 and SUV_mean_: 0.84), resulting in a TBR greater than 3, which was statistically significant compared to tumor activity (*p* ≤ 0.002 each) [46]. In this study, FAPI PET/CT showed a good application prospect in most primary and metastatic colorectal cancer (especially lymph node and liver metastasis). Compared to FDG PET/CT, the SUVmax of FAPI was higher, which was more beneficial to the discovery of lesions (Table 2).

### 4.3. FAPI vs. FDG Imaging in Colorectal Cancer

At present, ^18^F-FDG PET/CT is widely used in the disease management and treatment strategy of colorectal cancer to monitor metastasis [48,49,50]. Glucose metabolism is a key factor in the development and progress of cancer, and the uptake of ^18^F-FDG is related to glucose metabolism [51]. However, with the development of targeted medicine and the increase of knowledge about the biology of colorectal cancer, it has been found that tumor heterogeneity leads to more aggressive tumor growth and negatively affects treatment response and patient survival [52]. Meanwhile, the existence of tumor heterogeneity may affect the glucose consumption and the uptake of ^18^F-FDG [53]. In addition, ^18^F-FDG PET/CT also has certain limitations. Glucose metabolism is characteristic of many diseases, resulting in poor specificity and organ physiological distribution of ^18^F-FDG (eg. in the liver), resulting in low TBR of the image, which affects the diagnosis. Because colorectal cancer is prone to liver metastasis and metastatic lesions may be heterogeneous, FDG may be restricted in detecting colorectal cancer metastatic lesions, especially liver metastasis. The emergence of FAPI PET/CT may remedy the defect of FDG PET/CT. The uptake of FAPI PET/CT in normal brain, liver and oral mucosa is low and has a better diagnostic effect and lower background noise when monitoring primary and/or metastatic lesions [54,55,56,57]. FAPI PET/CT may provide superior visualization for colorectal, liver, and breast cancers, among others.

**Figure 2:**
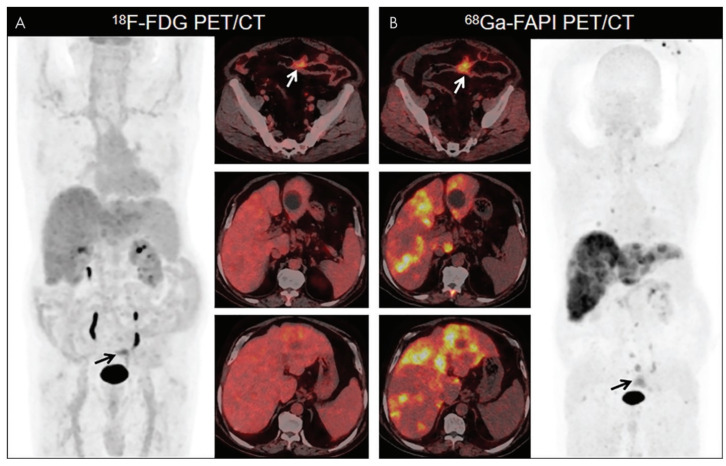
The uptake of ^68^GA-FAPI in the primary and metastatic lesions of colon cancer patients was higher than that of ^18^F-FDG, and the lesions were clearly shown. The black and white arrows show primary lesions of colon cancer. ^18^F-FDG PET/CT shows low to moderate metabolic activity in the primary colon and liver metastases ((**A**), left image: anterior maximum intensity projection image from ^18^F-FDG PET; right images: axial fused PET/CT images). ^68^Ga-FAPI PET/CT showed high uptake of primary lesions of colon and metastatic lesions of liver ((**B**), right image: anterior maximum intensity projection image from ^68^Ga-FAPI PET; left images: axial fused PET/CT images). Adapted from Ref. [59].

^68^Ga-FAPI PET/CT is a potential examination tool for disease management and treatment strategies of cancer. Compared to FDG, FAPI PET was more accurate for the overall staging and restaging, and FAPI PET altered clinical management in more than 20% of patients [58]. In the study of primary and recurrent gastrointestinal tumors, the detection rate of ^68^Ga-FAPI PET/CT for primary tumors was higher than that of ^18^F-FDG PET/CT, and the detection rates were 100% and 53% respectively (11 cases of gastric cancers and 9 cases of colorectal cancers). In addition, ^68^Ga-FAPI PET/CT showed that the tumor outline was clearer and the contrast of tumor background was higher. It was found that the lesions of primary colorectal cancer showed high uptake in ^18^F-FDG PET/CT and ^68^Ga-FAPI PET/CT, but the semi-quantitative parameter SUV_max_ was found in the analysis. The SUV_max_ of ^68^Ga-FAPI in these primary lesions was higher than that of ^18^F-FDG (15.9 vs. 7.9). In the assessment of recurrence, ^68^Ga-FAPI PET/CT had the higher true positive rates than that of ^18^F-FDG PET/CT (100% vs. 44%). On the contrary, the false positive rates of ^68^Ga-FAPI and ^18^F-FDG PET/CT for recurrence were different (11% vs. 2%). As a result, the specificity of ^68^Ga-FAPI was lower than that of ^18^F-FDG for patients in whom recurrence was detected (67% vs. 94%). In the evaluation of lymph node metastasis and organ metastasis of colorectal cancer, ^68^Ga-FAPI PET/CT detected more suspicious lymph nodes and organs than ^18^F-FDG PET/CT, and the uptake of ^68^Ga-FAPI was higher than ^18^F-FDG, showing high sensitivity (79% vs. 54%, respectively), while the specificity of ^68^Ga-FAPI PET/CT is not higher than that of ^18^F-FDG PET/CT (82% vs. 89%) [59] (Figure 2). ^68^Ga-FAPI PET/CT is obviously superior to ^18^F-FDG PET/CT in the display and image quality of peritoneal metastases of colorectal cancer (Table 3), and the average SUV_max_ value is obviously higher [60]. Moreover, in most studies, FAPI PET/CT is superior to FDG PET/CT in sensitivity and specificity for primary and metastatic colorectal cancer (especially lymph nodes and liver metastases), but the number of related studies and included patients is still relatively small. Further research will further understand the diagnostic performance of FAPI PET/CT for better clinical application. 

A 23-year-old female patient, ^68^Ga-FAPI-04 PET/CT showed that ^68^Ga-FAPI-04 was intensely taken up by lesion in the sigmoid colon, the lymph nodes in the retroperitoneum and the left supraclavicular fossa, and the pelvic abdominal peritoneum. Pathologically, it was confirmed as signet ring cell carcinoma of the sigmoid colon with peritoneal metastasis. The lesions were delineated more clearly by ^68^Ga-FAPI-04 scan, and more lesions were detected comparing to ^18^F-FDG scan [61]. Due to liver physiological distribution of ^18^F-FDG, ^18^F-FDG PET/CT may not be able to detect metastatic tumors or small metastatic tumors with low FDG uptake.

For the detection of colorectal cancer recurrence, compared to FDG, ^68^Ga-FAPI-04 PET/CT was found to have advantages in detecting recurrent signet ring cell colon cancer. A 36-year-old woman after surgery for left colon cancer, ^18^F-FDG PET/CT showed mild heterogeneous FDG uptake in the left perirenal soft tissue density lesions, with an SUV_max_ of 2.2, and an intense FAPI uptake in ^68^Ga-FAPI-04 PET/CT with an SUV_max_ of 12.8 [62]. 

Previous studies have confirmed that FAPI PET/CT detected more suspicious lymph nodes and organs than FDG PET/CT, which was more sensitive for identifying metastasis and recurrence in colorectal cancer, especially lymph node metastasis and organ metastasis. Based on a small sample study, 68Ga-FAPI PET/CT changed tumor or radiation tumor management in approximately 73.3% (11/15) of patients with metastasis [46]. However, the number of patients included in most current studies was relatively small, and most of them were in the advanced clinical stage. For advanced patients, even if FAPI PET/CT shows more lesions, the clinical stage will not change, so the therapeutical plan will remain the same. In terms of re-staging, FAPI PET/CT has more advantage than ^18^F-FDG, which provides key evidence for changing the clinical management of patients. This indicates that FAPI PET/CT may be more suitable for monitoring recurrence and metastasis of colorectal cancer patients and evaluating the therapeutical results. Therefore, it may be more meaningful for FAPI PET/CT to guide therapeutical decision-making and patient management to include more patients with lower clinical stage or monitor patients with recurrent colorectal cancer in future studies [58]. In addition, the physiological uptake of ^18^F-FDG in the liver restricts the application of FDG PET/CT in the detection of liver metastasis of colorectal cancer [63]. Encouragingly, FAPI PET/CT could help to improve the depiction of liver metastasis as a result of the clear tumor delineation and high TBR.

Compared to FDG, FAPI PET/CT has advantages in detecting colorectal cancer metastasis, even in small metastatic lesions. However, the diagnostic performance of FAPI PET/CT may be exaggerated due to the deviation of patient distribution. Therefore, appropriate imaging agents should be selected according to the patient’s conditions. The combination of two imaging agents can provide better information for patients help guide follow-up clinical oncologic management.

## 5. Limitations of FAPI PET/CT Imaging

**Figure 3:**
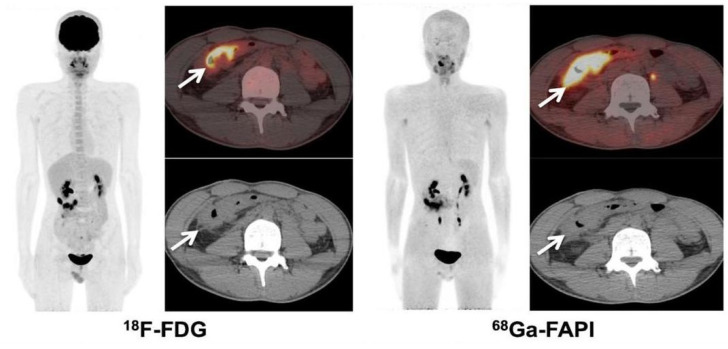
^18^F-FDG PET/CT showed hypermetabolism in the intestines around the anastomosis in the right upper quadrant of the abdomen (SUVmax 11.4). The involved bowels also had intense uptake of ^68^Ga-FAPI (SUVmax 14.1). The arrow shows high metabolic uptake of the intestinal tract around the anastomotic site after Crohn’s disease. Adapted from Ref. [71].

Research indicates that FAPI PET/CT may be a promising imaging method of improving the staging and treatment of colorectal cancer. Compared to FDG PET/CT, FAPI PET/CT has higher sensitivity in diagnosing the primary lesions of colorectal cancer and recurrence of colorectal cancer. However, it is also necessary to consider FAPI positive uptake due to inflammation-induced nonspecific fibrosis [64,65,66,67,68,69], which must be carefully distinguished. A recent study showed that FAP was also positive in Crohn’s disease [70]. A 28-year-old male with an 8-year history of Crohn’s disease underwent ^68^Ga-FAPI PET/CT imaging, which showed that there was an intense ^68^Ga-FAPI uptake in the diseased intestine (SUVmax, 14.1) [71]. However, in another patient with ulcerative colitis reported in this paper, there was only a phased increase in ^18^F-FDG intake consistent with the severity of the disease but no ^68^Ga-FAPI intake (Figure 3). This shows that we may obtain more and more valuable information on pathological tissue by combining different molecular probes, such as FDG, and analyzing the nature of pathological changes from multiple angles by integrating different information. Another 28-year-old woman with abdominal pain, intestinal dysfunction and weight loss for 3 months, ^68^Ga-FAPI PET/CT showed intense ^68^Ga-FAPI uptake in the transverse colon (SUVmax, 18.2) and a large number of abnormal lesions in the peritoneum as well as abnormal FAPI uptake (SUVmax, 10.6). Finally, this patient underwent colonic mucosa biopsy via endoscopy. The biopsy results showed granulomatous inflammation as well as acid-fast bacilli. The TSPOT.TB test (+). As a result, the patient suffers from intestinal tuberculosis [72]. These cases indicate that inflammatory enteritis may also be related to intense FAPI intake, which should be differentiated from colorectal cancer. Perhaps combined with the patient’s clinical symptoms and relevant medical history or relevant examination information, it can help us distinguish the nature of lesions with high FAPI uptake. As FAP is expressed on activated fibroblasts, when organ fibrosis occurs, the expression of FAP will increase and it will show high uptake in the subsequent FAPI PET/CT, which may be one of the reasons for false positive of FAPI PET/CT. Therefore, further exploring the FAPI PET/CT features of benign lesions characterized by fibrosis, combined with imaging information such as FDG PET/CT, may further improve the accuracy of FAPI PET/CT in the diagnosis of malignant tumors.

## 6. Discussion

Colorectal cancer is one of the main diseases that harm human health. Compared with FDG, FAPI PET/CT has advantages in detecting colorectal cancer metastasis, even in small metastatic lesions. However, the diagnostic performance of FAPI PET/CT may be exaggerated due to the deviation of patient distribution. However, FAPI PET/CT still provides a new perspective for the diagnosis and treatment of colorectal cancer.

Compared with FDG PET/CT, FAPI PET/CT has advantages in the diagnosis of primary tumors, various metastatic and recurrent lesions of colorectal cancer, providing important molecular imaging information for early diagnosis, accurate staging, guiding therapy, curative effect monitoring and recurrence evaluation. In addition, FAPI PET/CT may have special significance for delineating the target area of tumor radiotherapy. FDG PET mainly depends on the glucose metabolism of tumor cells to display the metabolic information of tumors and describes the shape of tumor by combining with the structural information of CT images. In solid tumors, apart from tumor cells, there is also a large amount of extracellular matrix. FAPI PET/CT can display the extracellular matrix by targeting fibroblasts in the tumor microenvironment, which may have special advantages in the morphological description of solid tumors. Therefore, the combination of FDG PET/CT and FAPI PET/CT image information may provide more useful information on tumor morphology and metabolism. 

At present, the clinical studies on FAPI PET/CT are still in the early stage, and most of them are small samples lacking long-term follow-up data, and false positive results caused by inflammation may restrict the application of FAPI PET/CT. These problems also require systematic clinical research on a large number of patients. At present, immunohistochemical staining has been used to correlate FAP expression with FAPI uptake in lesions, trying to further clarify their internal relationship, but related research is still limited, especially on the difference in FAPI uptake between malignant and non-malignant diseases. At the same time, because of the low expression of FAP in normal tissues, the strategy of targeting FAP has obvious advantages in tumor treatment, which is worth further study. 

## 7. Conclusions and Prospect

FAPI PET/CT, as a promising new PET imaging tool, may play an important role in tumor visualization and contribute to the diagnosis and staging of tumors. It is undeniable that FAPI PET/CT plays a unique role in the treatment of solid tumors, including colorectal cancer. Unfortunately, the exact mechanism and expression pattern of FAP in some diseases remain obscure, and the number of clinical studies on FAPI PET/CT is still small. Further research, including on its mechanism of action and more and larger-scale clinical studies, will help to clarify the true value of FAPI PET/CT in tumor and non-tumor diseases, which is of great significance to improving the diagnosis and treatment level. Although the research on FAPI PET/CT for colorectal cancer is still in the initial stage, we believe that with the deepening of research and the development of related technologies, FAPI PET/CT will be widely used in clinical applications.

## Figures and Tables

**Table 1:** Summary of major studies investigating FAPI PET/CT in colorectal cancer.

Author	Year	Type	Key Message
Loktev et al.	2018	Article	Cancer-associated fibroblasts are abundant in colon cancer and can be imaged using molecular probes targeting FAP.
Kratochwil et al.	2019	Article	FAPI PET/CT has intermediate strength uptake in colorectal cancer.
Koerber et al.	2020	Article	^68^Ga-FAPI PET/CT can detect primary and metastatic tumors of the lower digestive tract, identify clinical stages and provide evidence for tumor management.
Rathke et al.	2021	Case	FAPI uptake was high in colorectal cancer-related metastases, and peritoneal metastases disappeared after treatment with ^90^Y-FAPI46.
Giesel et al.	2021	Article	In the background tissues of colon, the SUVmax of ^68^Ga-FAPI was significantly lower than that of ^18^F-FDG.
Şahin et al.	2021	Article	^68^Ga-FAPI is superior to ^18^F-FDG in detecting liver metastasis of colorectal cancer.
Pang et al.	2021	Article	^68^Ga-FAPI was superior to ^18^F-FDG in the detection of colorectal cancer primary and metastatic sites, showing higher uptake in most primary and metastatic sites.
Fu et al.	2021	Case	^68^Ga-FAPI has strong uptake in sigmoid signet ring cell carcinoma and is more sensitive to primary lesions and peritoneal carcinomas than ^18^F-FDG.
Mona et al.	2022	Article	FAPI PET/CT has strong uptake in colorectal cancer patients, and the intensity of FAPI uptake is related to the intensity of immunohistochemical staining of FAP.
Strating et al.	2022	Article	FAPI PET/CT may have important potential value in evaluating CMS4 as an adverse prognostic factor for colorectal cancer.
Qin et al.	2022	Article	^68^Ga-FAPI is of great significance in the accurate staging and clinical management of colorectal cancer.
Elboga et al.	2022	Article	The uptake level of FAPI in primary and metastatic lesions of colorectal cancer (especially liver metastasis) was significantly higher than that of FDG and had a better tumor-to-background ratio.
Güzel et al.	2022	Case	^68^Ga-FAPI was superior to ^18^F-FDG in evaluating recurrent signet ring cell colon cancer.
Kömek et al.	2022	Article	^68^Ga-FAPI has higher sensitivity and specificity in detecting primary lesions of colorectal cancer and lymph node and peritoneal metastasis.

**Table 2:** SUVmax of FAPI and FDG in colorectal cancer lesions.

Author	Year	Lesion Location	FAPI ^#^ SUVmax	FDG SUVmax
Koerber et al.	2020	Primary lesions	15.7	-
		Local relapse	6.56	-
		Lymph node metastases	8.33	-
		Liver metastases	9.54	-
Şahin et al.	2021	Primary lesions	5.5	5.0
Pang et al.	2021	Primary lesions	15.9	2.2
		Lymph node metastases *	6.7	2.4
		Liver metastases *	9.7	1.7
Elboga et al.	2022	Primary lesions	14.6	8.4
		Lymph node metastases	9.9	4.9
		Peritoneal metastases	10.7	3.1
		Liver metastases	12.2	5.0
Kömek et al.	2022	Primary lesions	11.54	18.93
		Lymph node metastases	3.6	2.25
		Peritoneal metastases	5.14	3.59
		Liver metastases	6.15	9.66

^#^ FAPI: fibroblast-activating protein inhibitors. * The data included gastric, duodenal, and colorectal cancers.

**Table 3:** The sensitivity and specificity of FAPI and FDG in colorectal cancer lesions.

Author	Year	Lesion Location	FAPI ^#^ Sensitivity	FAPI ^#^ Specificity	FDG Sensitivity	FDG Specificity
Şahin et al.	2021	Liver metastases	96.6%	-	70.8%	-
Pang et al.	2021	Lymph node metastases *	79%	82%	54%	89%
Kömek et al.	2022	Primary lesions	100%	100%	100%	85.3%
		Lymph node metastases	90%	100%	80%	81.8%
		Peritoneal metastases	100%	100%	55%	100%

^#^ FAPI: fibroblast-activating protein inhibitors. * The data included gastric, duodenal, and colorectal cancers.

## Data Availability

Not applicable.
